# Experience-independent sex differences in newborn macaques: Females are more social than males

**DOI:** 10.1038/srep19669

**Published:** 2016-01-22

**Authors:** Elizabeth A. Simpson, Ylenia Nicolini, Melissa Shetler, Stephen J. Suomi, Pier F. Ferrari, Annika Paukner

**Affiliations:** 1 Department of Psychology, University of Miami, Coral Gables, Florida, USA; 2 Laboratory of Comparative Ethology, Eunice Kennedy Shriver National Institute of Child Health and Human Development, National Institutes of Health, Department of Health and Human Services, Poolesville, Maryland, USA; 3 Dipartimento di Neuroscienze, Università di Parma, Parma, 4300 Italy; 4 Unit on Computer Support Services, Eunice Kennedy Shriver National Institute of Child Health and Human Development, National Institutes of Health, Department of Health and Human Services, Bethesda, Maryland, USA

## Abstract

Human females exhibit greater social interest and skills relative to males, appearing in infancy, suggesting biological roots; however, male and female infants may be treated differently, potentially causing or amplifying sex differences. Here, we tested whether sex differences in social motivation emerge in infant monkeys (*n* = 48) reared in a controlled postnatal environment. Compared to males, females at 2–3 weeks looked more at conspecifics’ faces (*d* = 0.65), especially the eyes (*d* = 1.09), and at 4–5 weeks exhibited more affiliative behaviors (*d* = 0.64), including gesturing, looking, and proximity to familiar and unfamiliar human caretakers. In sum, converging evidence from humans and monkeys suggests that female infants are more social than males in the first weeks of life, and that such differences may arise independent of postnatal experience. Individual differences in social interest have wide-ranging developmental consequences, impacting infants’ social interaction quality and opportunities for learning. Understanding the evolution of sex differences and their developmental emergence is necessary to best support infants with varying levels of sociality.

In humans, sex differences appear at the level of the brain, cognition, and behavior^1^,^2^, across numerous domains, including physical and mental health^3^,^4^, personality^5^, and sexuality^6^. Females, compared to males, exhibit greater social sensitivity^7^ and stronger verbal ability^8^, while males outperform females on mental rotation^9^ and the analysis or construction of systems^10^. Sex differences in social behavior are already evident in infancy^11^. Female neonates, compared to males, make more eye contact^12^, are more likely to orient to faces^13^ and voices^14^, are rated as more cuddly^15^, and exhibit stronger emotion contagion (e.g., contagious crying^16^) and imitation^17^. Despite converging evidence of sex differences in social sensitivity early in ontogenetic development, the causes of these differences, and contributions of early experience, remain unresolved.

According to one view, sex differences may, at least in part, be a consequence of evolutionary pressures, reflecting a history during which males and females faced different challenges for survival and reproduction^18^. According to this perspective, selective pressures may partially explain some of these sex differences^19^,^20^. For example, across most mammals, females are the primary caretakers, a role that may have increased females’, but not males’, social interest and skill interpreting nonverbal expressions, as such interests and skills, in theory, might increase offspring survival, preparing caregivers to recognize and respond to infants’ needs^21^,^22^,^23^. While such evolutionary proposals remain to be fully tested, they are consistent with the evidence to date.

Complicating matters, however, are reports that male and female infants are treated differently from the day of birth and throughout infancy^24^,^25^,^26^; therefore, differential early caregiver or other environmental stimulation may cause or contribute to sex differences. For example, in the first months of life, males are touched more and handled more roughly^27^, but females are verbally stimulated more^28^, and mothers spend more time in synchronous coordination with sons^29^,^30^, but interact more overall with daughters^31^. Even if such differential treatment is small^32^, it may nonetheless contribute to different socialization^33^. The extent to which sexually dimorphic behaviors reflect inborn or natural biological differences, independent of parental influence, remains untested.

Nonhuman primate (NHP) studies can begin to address this challenge^34^,^35^, as there is greater control over NHPs’ early experiences, potentially eliminating postnatal environmental causes of sex differences. In addition, macaques, like humans, are highly social and engage in complex face-to-face infant-adult interactions^36^. Newborn macaques possess good visual acuity and we can assess their visual attention with remote eye tracking^37^,^38^. Infant and juvenile macaques exhibit sex differences in a variety of behaviors—rough-and-tumble play, peer preferences, social grooming, and infant interest—that parallel sex differences in humans^39^,^40^. As in humans, macaque mothers treat male and female infants differently, for example, grooming female infants more than males and responding more to males’ separation vocalizations^41^,^42^,^43^. In fact, macaques are one of the only species, besides humans, in which the early social environment has been shown to influence sex differences in behavior^44^. However, no study, to date, has controlled human or NHP infants’ environment from birth; therefore, the extent to which sex differences are experience-independent, or due to infants’ early experiences, have yet to be explored.

In this study, we assessed sex differences in nursery-reared macaque infants, raised in homogenous, controlled environments (see Suppl. Info.). Remote eye tracking revealed that females, compared to males, looked more at videos of expressive conspecific faces and especially the eye region (Fig. 1) at 2 to 3 weeks of age. Furthermore, in a human interaction test, females displayed more affiliative behaviors (e.g., facial gestures, close proximity) to familiar and unfamiliar social partners, compared to males when 4 to 5 weeks old. In sum, in the absence of different postnatal environments, across two tasks, females appeared more social than males. While the long-term consequences of these individual differences are currently unknown in macaques, in humans, diminished social motivation in infancy may signify individuals at risk for poor developmental outcomes^45^. Our results offer compelling evidence that, through this novel approach, we can begin to disentangle biological (postnatal experience-independent) and experiential influences on sexually dimorphic behaviors, such as social interest.

## Results

### Eye Tracking Test

We first carried out a 3 × 3 × 2 mixed design ANOVA on look durations to the face, with the within-subjects factors of Expression (Fear, Lipsmacking [LPS], Threat) and Phase (Expression, Still, Turn), and the between-subjects factor of Sex (Female, Male). There was a main effect of Phase, *F*(2,60) = 12.40, *p* < 0.001, η_p_^2^ = 0.246, in which infants looked more during the period of Expression (*M* = 2.34, *SD* = 0.65) and Turn (*M* = 2.29, *SD* = 0.76), compared to Still (*M* = 1.98, *SD* = 0.64), *t*(47) > 4.19, *p*s < 0.001, *d*s > 0.61. There was a main effect of Sex, *F*(1,38) = 6.58, *p* = 0.014, η^2^ = 0.148, in which females looked more (*M* = 2.41 sec, *SD* = 0.55) than males (*M* = 2.03 sec, *SD* = 0.61), Fig. 2a. There were no other effects, *p*s > 0.05.

We next carried out a 3 × 3 × 2 mixed design ANOVA on the Eye-Mouth-Index (EMI), with the within-subjects factors of Expression and Phase, and the between-subjects factor of Sex. This analysis revealed a main effect of Phase, *F*(2,60) = 10.10, *p* < 0.001, η_p_^2^ = 0.252, in which there was a lower EMI (more looking to the mouth) for the periods of Expressions (*M* = 0.69, *SD* = 0.17) compared to either Still (*M* = 0.81, *SD* = 0.12) or Turn (*M* = 0.79, *SD* = 0.19), *t*(47) > 3.75, *p*s ≤ 0.001, *d*s > 0.54. There was a main effect of Sex, *F*(1,30) = 7.07, *p* = 0.012, η^2^ = 0.191, in which females had higher EMI (*M* = 0.83, *SD* = 0.06) compared to males (*M* = 0.70, *SD* = 0.14) (Fig. 2b; Fig. S1). There were no other effects, *p*s > 0.05.

### Human Interaction Test

We carried out three 2 × 2 mixed-design ANOVAs, one on each composite measure—Affiliative Social, General Arousal, and Stress/Anxiety—with the between subjects factor Sex and the within subjects factor of Person Type (Stranger, Familiar), Fig. 3. The ANOVA on Affiliative Social revealed a main effect of Sex, *F*(1, 46) = 5.04, *p* = 0.030, η^2^ = 0.099, in which female infants were more social (*M* = 0.23, *SD* = 0.62) compared to males (*M* = −0.18, *SD* = 0.68). There were no main effects of Sex for either General Arousal nor for Stress/Anxiety, *F*(1, 46) = 0.022, *p* = 0.882, and *F*(1, 46) = 0.016, *p* = 0.899, respectively. There were no main effects or interactions for the factor Person Type for any of the composite measures (Social/Affiliation: *F*(1,46) = 0.031, *p* = 0.861, *F*(1,46) = 1.987, *p* = 0.165; General Arousal: *F*(1, 46) = 0.002, *p* = 0.964, *F*(1,46) = 0.129, *p* = 0.721; Stress/Anxiety: *F*(1, 46) = 0.001, *p* = 0.976, *F*(1, 46) = 0.173, *p* = 0.680, respectively).

## Discussion

There is considerable variability in infants’ social interest^37^. One factor that seems to predict infants’ sociality is sex^7^,^11^,^12^,^13^,^14^,^15^,^16^,^17^; however, the causes of these sex differences and the role of the early environment, in particular, have yet to be uncovered. Here, we tested whether infants reared in controlled homogenous environments from birth would still exhibit sex differences in social interest. We found consistent sex differences in infants’ social interest: females, compared to males, exhibited greater social interest and affiliative behavior. These results are striking because infants were reared in carefully controlled environments, making environmental (e.g., caregiving) causes, theorized to account for sex differences in humans^24^,^25^,^26^,^27^,^28^,^29^,^30^,^31^,^32^,^33^, unlikely. An internal quality assessment of caregiver training protocols confirmed that caregivers were not more sensitive to female than male infants (see Suppl. Info.). Note that we do not want to make any claims as to the generalizability of this observation; rather, we believe it shows that the specific training protocols at this facility were effective in preventing caregiver bias. Thus, it is unlikely that our findings of greater social interest among females can be attributed to differences in caregivers’ behavior. The present study is the first (in any primate species, including humans) to provide evidence of *experience-independent* sex differences in sociality present or emerging soon after birth.

Caregiver-infant interactions are complex and multimodal, varying across cultures and contexts: some occurring primarily through tactile stimulation (e.g., holding, patting, stroking), while others rely more on visual (e.g., mutual gaze, facial gestures) or verbal interactions^36^,^46^,^47^. Thus, infant sociality can be expressed in different ways across various cultural contexts. Despite this variability, certain key features appear universal in human and nonhuman primate infants, including an early attraction to faces^38^. Here, we found sex differences in sociality across two tasks. First, in an eye tracking task, in which 2- to 3-week-old monkey infants viewed affiliative, fearful, and threatening monkey facial expressions, females, compared to males, spent more time looking at faces, and spent a greater proportion of time looking to the eyes. Similarly, in a human-interaction task, in which 4- to 5-week-old monkey infants were presented with unexpressive human models attempting eye contact, females, compared to males, engaged in more affiliative behaviors towards both familiar and unfamiliar humans. Together, these results suggest that macaque infants in controlled postnatal environments exhibit sex differences in social interest and affiliation, with females appearing more interested in social interactions than males. Our data suggest that such differences are unlikely to be exclusively due to different postnatal environments, as the postnatal environment was controlled in the present study. Rather, there appear to be experience-independent sex differences in social behavior in early infancy, and the present results begin to reveal the nature of these sex differences that are not due to social experiences. While the present study does not rule out the possibility that experiences may also contribute to sex differences—and we agree with others^24^,^25^,^26^ that they likely do—it suggests that differential experiences are unnecessary for the initial expression of sex differences in social behaviors in infant monkeys. These data provide additional support for the hypothesis that sex differences in social behavior can arise independent of social mechanisms^48^.

Our data are consistent with reports in human infants that females are drawn more to biological motion and faces compared to males^13^,^17^,^49^,^50^. The present study did not include nonsocial control stimuli, which may be more engaging for males; future assessments that include social and nonsocial stimuli presented in direct competition^51^ could help clarify sex differences in infants’ relative visual interest. Nonetheless, the present paradigm revealed female infants, compared to males, looked longer at facial expressions, suggesting females may find faces intrinsically more rewarding.

Our results are also consistent with findings in human infants that females, compared to males, spend more time in eye contact^12^,^25^, a difference that persists through childhood and into adulthood^52^. In the present study, this sex difference may reflect the fact that eye contact is one of the first ways in which infants can engage in social exchanges, which, in humans, is speculated to be foundational for later social skills^53^. Indeed, newborn monkeys who look more at the eye region of faces are also better at imitating facial gestures^37^, and imitation predicts later social skills, such as gaze following (i.e., the ability to look where another individual is looking)^35^,^54^.

In addition, differential parental behavior towards infants as a function of infant sex may, at least in part, stem from and amplify initial biological differences. Adult macaques—much like adult humans—differentially treat infants depending on their sex^41^,^42^,^43^,^46^; however, it is unclear how this differential treatment may impact infants’ early social interest. Future studies in infant NHP may be fruitful in this regard, as they allow us to explore the extent to which natural maternal interactions or other specific aspects of infants’ early social experiences may drive or dampen early sex differences. Further work is needed on these potential feedback loops and interactions; conclusions about causality are therefore premature at present.

While studies in human infants have found that females are better at discriminating facial expressions than males^55^, we did not find any differential looking across our facial expression types. This may be because 2- to 3-week-old infant monkeys do not understand these expressions until around 2 to 3 months of age^56^. While these infants had previously seen human models lipsmacking, in unrelated studies (see Methods), they had no exposure to adult monkeys producing these expressions, nor did they have any previous exposure to open-mouth threat expressions or fear grimace expressions, as were shown here. Here we did not explicitly test facial expression discrimination, nor did we record infants’ other behavioral reactions beyond their viewing patterns (e.g., their emotional reactions or facial expressions). Many questions, therefore, remain regarding newborn emotion processing. For example, in human neonates, contagious crying—hypothesized to reflect an early form of empathy in infants—appears stronger in females than males^16^, but such assessments have yet to be carried out in infant NHP.

Our finding that female macaque infants, compared to males, exhibited more social and affiliative behaviors towards both familiar and novel human models suggest that female infant monkeys are more interested in social interactions compared to males. We found no differences in their general arousal (e.g., sleepiness) or behaviors indicative of stranger-anxiety (e.g., self-directed behaviors), which could have been alternative explanations for the observed effects. Nonetheless, these data seem consistent with reports in human infants. In humans, female infants, compared to males, are more responsive to their mother’s voice, initiate more maternal social interactions, and spend more time in close proximity to their mothers^57^,^58^. In addition, human 3-month-old females smile more than males while interacting with strangers in face-to-face interactions^59^.

In the human interaction task, the human produced a neutral face, attempting to maintain eye contact with the infant. Although speculative, it is possible that male infants may have been more likely to interact had the human initiated the interaction with a communicative gesture or, at the least, if the human had appeared more responsive to the infant’s interaction attempts. One interpretation of our results is that it may take a more engaging adult partner to attract male infants’ interest relative to females. For example, when mothers were instructed to direct fearful expressions at their infants in a social referencing task (i.e., infants had to use their mother’s expression to respond to an ambiguous situation), mothers’ expressions were less intense when directed at female infants compared to male infants, perhaps reflecting the mothers’ awareness of their infants’ sensitivity to such expressions^60^. Thus, this human interaction task seems to assess some combination of infants’ ability, interest, and persistence in initiating a social interaction, even one that appears failing. A similar task in human infants is the still-face paradigm, in which a parent interacts with the infant normally and then produces an unresponsive still-face^61^. We are unaware of any reports that female infants try harder to re-engage parents in social interactions during this still-face test, as they appeared to do in the present study; however, in one report female infants did appear more distressed than males^61^.

In humans, mother-stranger discrimination has been reported to occur earlier in female infants, compared to males, possibly reflecting faster social development in female infants^62^. We also expected differences in infants’ reactions to familiar compared with novel human models. However, infant monkeys do not generally exhibit fear of strangers or novelty until 2.5- to 3-months-old; here, infants may have been too young to exhibit noticeably different responses to familiar and unfamiliar social partners, at least in this context.

In summary, infant monkeys appear to exhibit experience-independent sex differences in the first month of life, with female infants, compared to males, displaying more visual attention and affiliative behaviors towards social stimuli, including increased gaze to faces and especially the eyes, facial gestures, proximity, and touch. The present study is not without limitations, however. We were unable to completely rule-out other potential causes of sex differences, such as more subtle differential treatment by caregivers or research staff, especially beyond 3 weeks of age. However, we think these are unlikely to account for the present findings for two reasons. First, we found infant sex differences within the first 3 weeks of life, so even if infants are treated differently after 3 weeks that cannot account for the present findings. Second, infants participated in only one test with research staff prior to this study (see Suppl Info.), making it unlikely that differential treatment during this standardized interaction is responsible for our findings. Further observations assessing subtler differential treatment of infants, however, are a worthy future direction. Another challenge that needs to be addressed in future work is how to disentangle social skill from social motivation^45^, because without the later, the former cannot be assessed. Making tasks equally engaging for male and female infants may be difficult, but is nonetheless critical for fairly assessing possible differences in social skills. Finally, the extent to which these findings are generalizable to other cultural contexts, or predictive of social outcomes at later ages, is yet to be determined.

Newborns’ early capacities to engage with social partners—including their interest in faces, eye-contact, and other affiliative expressions (e.g., facial gestures)—provide an early window which may ultimately be useful for understanding individual differences and predicting developmental trajectories^47^. Visual attention to social stimuli seems particularly promising in this regard^63^. Early sex differences may be related to later behaviors, including sex differences in developmental disorders and disabilities^24^,^64^. Finally, our findings are consistent with evolutionary hypotheses about the origin of sex differences in social behavior^20^, possibly reflecting an evolved mechanism enabling the care of nonverbal infants, ultimately increasing infant survival (i.e., primary caretaker hypothesis^21^). Determining specific causes of sex differences necessitates further study at multiple levels, including proximate and ultimate causes and their interactions. Nonetheless, studying sex differences across development in humans and NHP in controlled environments may provide important insights into the evolution of sex differences.

## Method

### Subjects

Subjects were 48 healthy, full-term infant rhesus macaques (*Macaca mulatta*). For the eye tracking task, we tested infants at 2–3 weeks (10–28 days old), including 21 females (*M* = 18.9 days, *SD* = 2.2) and 27 males (*M* = 18.7 days, *SD* = 2.5). For the human interaction task, we tested infants again at 4–5 weeks (28–37 days old), including 21 females (*M* = 31.7 days, *SD* = 2.0) and 27 males (*M* = 31.4 days, *SD* = 1.9). Infants were separated from their mothers on the first day of life, after which they were reared in a nursery facility. Infants were tested prior to introduction into social groups with conspecifics. Human caretakers and research staff followed strict protocols ensuring male and female infants were not treated differently. All infants participated in unrelated studies that involved structured social interactions with humans in the first week of life, including neonatal imitation^36^; because these were structured, they were preformed in the same way for all infants. For details, see the Suppl. Info. The study was approved by the Animal Care and Use Committee, conducted in accordance with the Guide for the Care and Use of Laboratory Animals, and complied with the Animal Welfare Act.

### Materials and Procedure

Eye movements were recorded via corneal reflection using either a Tobii T60XL (*n* = 38) or a Tobii TX300 (*n* = 10) eye tracker, with a remote 61 cm and 58.4 cm monitor, respectively, both with integrated eye tracking technology and a sampling rate of 60 Hertz. We used Tobii Studio software (Tobii Technology, Sweden) to collect and summarize the data.

At 2–3 weeks of age, infants viewed three silent video stimuli, depicting an animated adult monkey looking at infants and exhibiting either LPS (an affiliative gesture), fear grimaces, or threats (see Suppl. Info.). The macaque, making eye contact with the viewer, displayed a 5 sec expression (fear grimaces, LPS, or threats), followed by a 5 sec neutral face (eye blinks and small head movements were included to maintain an animated impression). Then the macaque turned away at a 45° angle, breaking eye contact, and then turned back to the viewer. This sequence was repeated a second time, for a total duration of 30 sec.

At the beginning of a session, an experimenter held the infant approximately 60 cm from the screen. Each infant was calibrated to Tobii Studio’s five preset locations. Infants were tested with one video per day. Videos were shown in a random order.

At 4–5 weeks of age, infants participated in a human interaction task. A human model was seated in front of the infant’s home cage, 30 cm from the cage front, and made eye contact with the infant. During the first 2 minutes of the test, the human model only looked at the infant. During the second 2 minutes of the test the human placed a hand on the infant’s feeder box, located just outside of the infant’s home cage, while continuing to maintain eye contact. Sessions were videotaped (Sony Digital Video HDR-CX560V) with only the infant in view. In total, each session was 4 minutes. We were primarily interested in social behaviors, but also assessed general arousal and anxiety-related behaviors (e.g., self-directed behaviors). In total, we scored 15 behaviors, including affiliative social behaviors: LPS and tongue protrusion facial gesture frequencies, total time looking at model, time touching model’s hand, time in close proximity to model (within arm’s reach of font of cage). Two coders scored behaviors using The Observer XT (Noldus). See Suppl. Info. for details.

### Data Analysis

**Figure 1:**
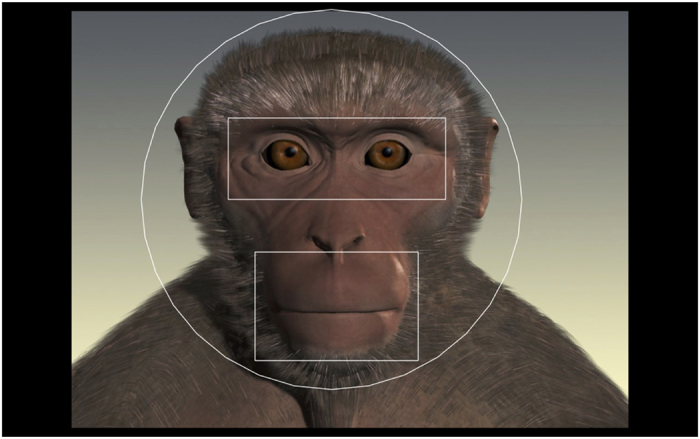
Face, Eye, and Mouth areas of interest (AOIs) on monkey avatar. The third author, Melissa Shetler, created this video stimulus.

**Figure 2:**
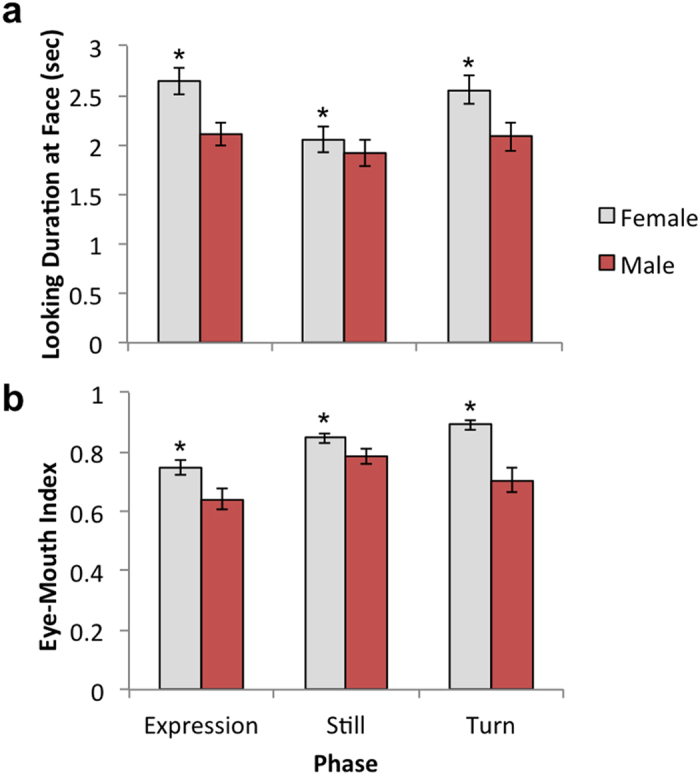
Eye tracking task results. Across both measures, there were main effects of sex: females (gray) > males (red), *ps < 0.05. Error bars reflect standard error of the mean. (**a**) Look durations to faces. (**b**) The eye-mouth index (EMI) reflects the relative amount of time looking to the eye and mouth regions of the face, with values of .50 indicating equal looking to eyes and mouth, and values above .50 indicating more looking to the eyes.

**Figure 3:**
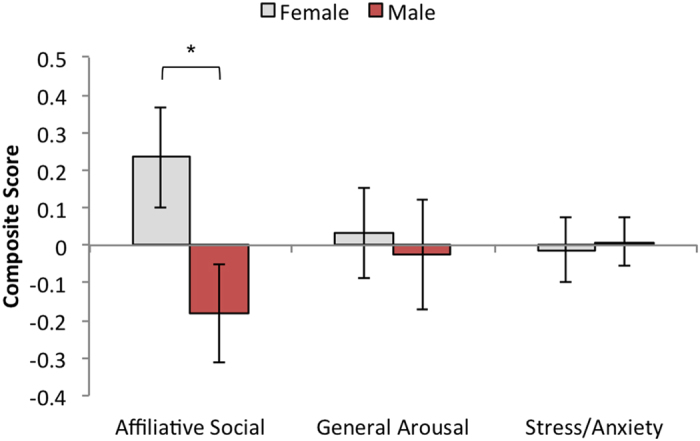
Human interaction task results. Composite (standardized) scores are graphed (*M* = 0; *SD* = 1). There was a sex differences for the Affiliative Social composite: females (grey) > males (red), *p = 0.034. There were no other differences, *p*s > 0.05. Error bars reflect standard error of the mean.

In Tobii Studio we created several Areas of Interest (AOIs) for analysis: Face, Eye, and Mouth AOI (see Fig. 1 and Suppl. Info.). We created an Eye-Mouth-Index (EMI) using Eyes / (Eyes + Mouth) in order to compare looking to both areas^37^. Values closer to 1 indicate more looking to the eyes, and values closer to 0 indicate more looking to the mouth.

For the human interaction task, we computed three composite scores by standardizing then averaging individual behavior scores. The Affiliative Social composite included facial gestures, and looking, touching, or being in close proximity to model. The General Arousal composite included exploration, locomotion, and sleeping. The Stress and Anxiety composite included scratching, fear grimacing, vocalizing, clinging to surrogate, self-clasping, self-sucking, and stereotypies. Interobserver reliability was high (see Suppl. Info.).

### Ethical approval

Research methods were approved by the Animal Care and Use Committee, *Eunice Kennedy Shriver* National Institute of Child Heath and Human Development, National Institutes of Health (ASP#11-043 and #14-043). The study was conducted in accordance with the Guide for the Care and Use of Laboratory Animals and complied with the Animal Welfare Act.

## Additional Information

**How to cite this article**: Simpson, E. A. *et al*. Experience-independent sex differences in newborn macaques: Females are more social than males. *Sci. Rep.* **6**, 19669; doi: 10.1038/srep19669 (2016).

## Supplementary Material

Supplementary Movie 1

Supplementary Movie 2

Supplementary Movie 3

Supplementary Information
