# Supplementary material for: Experience-independent sex differences in newborn macaques: Females are more social than males
Source: Sci Rep. 2016 Jan 22;6:19669. doi: 10.1038/srep19669 (PMC4726418; doi:10.1038/srep19669)
Supplement: Supplementary Information [file srep19669-s4.doc]

**Experience-independent sex differences in newborn macaques: Females are more social than males**

Short title: Experience-independent sex differences

Elizabeth A. Simpson ^1,2,3^,*^,A^, Ylenia Nicolini^3^, Melissa Shetler^4^, Stephen J. Suomi^2^, Pier F. Ferrari^3^, and Annika Paukner^2,A^

1. Department of Psychology, University of Miami, Coral Gables, Florida, USA
2. Laboratory of Comparative Ethology, *Eunice Kennedy Shriver* National Institute of Child Health and Human Development, National Institutes of Health, Department of Health and Human Services, Poolesville, Maryland, USA
3. Dipartimento di Neuroscienze, Università di Parma, Parma, 4300 Italy
4. Unit on Computer Support Services, *Eunice Kennedy Shriver* National Institute of Child Health and Human Development, National Institutes of Health, Department of Health and Human Services, Bethesda, Maryland, USA

*Corresponding author. Department of Psychology, University of Miami, 5665 Ponce De Leon Drive, Coral Gables, Florida 33146. Phone: +1-305-284-6181. Email: simpsone@miami.edu.

^A^ E. Simpson and A. Paukner contributed equally.

Supplementary Information

**Methods**

**Subjects.** Infants were individually housed for the duration of these experiments. They were housed in incubators (51 cm × 38 cm × 43 cm) for the first two weeks of life and in metal cages (61 × 61 × 76 cm) thereafter. Both housing arrangements contained an inanimate surrogate covered with fleece fabric as well as loose pieces of fleece fabric and rubber toys. Infants could see and hear, but not physically contact, other infants of similar age. While we did not formally quantify social interactions between infants in adjacent ages, such interactions were rare and typically brief (e.g., one infant looking at another for a few seconds). Importantly, these opportunities were equal across male and female infants, and therefore cannot account for the present findings.

Human caretakers, present for 13h each day, interacted with infants every 2h for feeding and cleaning. While it was not possible for caretakers to be blind to infant sex, we took steps to minimize the influence of infant sex. Animal care staff and research staff followed strict protocols ensuring standardized treatment of all infants. A recent internal quality assessment of caregiver interactions with infants aged 0-3 weeks old confirmed that caretakers did not treat male infants any different from female infants (see below). In addition, infants were assigned identification numbers at birth instead of identifying names.

**Materials and Procedure**

**Eye Tracking Task.** At 2-3 weeks of age, infants viewed three silent video stimuli (Supplementary Videos 1-3), depicting an animated adult monkey (head and shoulders) looking at infants and exhibiting either a fear grimace, lipsmack (LPS; a positive, affiliative gesture), or threat. Videos were created with Maya and Zbrush software. Screen and video resolution were set to 1280 x 800 pixels (Tobii T60XL), or 1280 x 720 pixels (Tobii TX300).

All infants were newly calibrated for each test session. Individual calibration points that were judged to be unreliable were repeated until an acceptable calibration was obtained. Infants were tested with only one video per day; 10 infants saw each video once and 38 infants saw each video twice.

**Human Interaction Task.** At 4 to 5 weeks of age, infants participated in a human interaction task^1^. We were primarily interested in social behaviors, but also assessed general arousal and anxiety-related behaviors (e.g., self-directed behaviors^2^). In total, we scored 15 behaviors: affiliative social behaviors—LPS and tongue protrusion facial gesture frequencies, total time looking at model, time touching model’s hand, time in close proximity to model (within arm’s reach of font of cage); behaviors that reflect more general arousal; time spent exploring (actively interacting with toys, enrichment), locomotion (moving > 30 cm), and sleeping (laying down); and behaviors that indicate stress or anxiety—frequency of scratching bouts, fear grimaces, vocalizations, and time spent clinging to surrogate, self-clasping, self-sucking (e.g., thumb), and engaging in stereotypic behaviors (e.g., rocking). These behaviors were combined into three composite measures (see Method and Tables S1-S2). Two coders scored behaviors offline, frame-by-frame, using The Observer XT (Noldus). Coders were blind to infant sex and condition (stranger, familiar).

**Data Analysis**

**Eye Tracking Task.** In Tobii Studio we created several Areas of Interest (AOIs) for analysis: Face, Eye, and Mouth AOI (see Fig. 3). The Face AOI was 700 x 700 pixel. Eye and Mouth AOIs were 400 × 150 pixel and 300 × 200 pixel respectively. We used Tobii Fixation Filter to extract total fixation durations for each AOI. Data were trimmed between subjects to remove outliers greater than two standard deviations from the mean. This resulted in the trimming of 3% of data for looking to the face, and 5% of data for the eye-mouth index (EMI).

**Human-Interaction Task.** We calculated interobserver agreement on 20% of sessions (19 videos), Table S1.

**Quality Assessment Data of Caregiver-Infant Interactions**. To ensure that caregivers followed the facility-specific infant care protocols and treated all infants in the same manner, an internal quality assessment was carried out on 16 infants (9 females, 7 males). Infants were observed daily for the first 21 days of life during routine care, including feeding and handling. Three observers, naïve to infants’ sex, observed 10 caregivers during 5-min focal observations, four times per day (~8am, 10am, 12pm, 2pm). Observers rated whether the caregiver was “sensitive to infant state” and the quality/quantity of “caregiver-initiated social interaction” on a scale from 1 “Very Low” to 5 “Very High” (with 3 labeled “Moderate”). In addition to these overall assessments, observers rated other specific caregiver behaviors (see list in Table S3), on a scale from 1 “Not at All” to 5 “Very Much” (with 3 labeled “Moderate”). The last rating was: “If held, rate overall roughness” on a scale from 1 “Very Gentle” to 5 “Very Rough.”

**Quality Assessment Analyses**. We first computed an Overall Caregiver Sensitivity composite measure based on 14 ratings (Table S3), each of which was converted to a *Z* score (with Roughness inverse coded), then averaged. We carried out a 2 × 3 mixed design analysis of variance (ANOVA) with the between-subjects factor Sex (Male, Female) and the within-subjects factor Week (1, 2, 3). This revealed a main effect of Week, *F*(2, 28) = 22.47, *p* < .001, η^2^ = .616, with greater caregiver sensitivity in the third week (*M* = .383, *SD* = .32) compared to the first two weeks (*M* = .053, *SD* = .10, and *M* = .003, *SD* = .11, respectively), *t*(15) > 4.94, *p*s < .001, *d*s > 1.23, but no difference between weeks 1 and 2, *t*(15) = 1.56, *p* = .178. There was no main effect of Sex, *F*(1,14) = 2.31, *p* = .151, nor a Week × Sex interaction, *F*(2,28) = 2.09, *p* = .143.

To further search for potential sex differences, we carried out a multiple analysis of variance (MANOVA) on the 14 caregiver ratings (Table S3) at each week, which revealed no effects of Sex, ps > .05. To explore potential Sex × Week interactions, we next carried out 2 × 3 ANOVAs on each of the 14 caregiver ratings (Table S3), with the factors Sex and Week. There were no main effects of, or interactions with, the factor Sex, for any measure (*p*s > .05), except one trend of a main effect of Sex for Stroking, *F*(2,14) = 6.26, *p* = .025, η^2^ = .309, in which males (*M* = 2.59, *SD* = .17) were stroked more than females (*M* = 2.35, *SD* = .21); this effect, however, was not retained with a correction for multiple comparisons (*p* = .05 / 14 comparisons = .004). In sum, there was no evidence that caregivers exhibited greater sensitivity with females compared to males, suggesting that facility-specific training protocols were effective in preventing caregiver bias.

These ANOVAs also revealed 8 main effects of Week (see Table S3 for descriptive statistics): Interaction Quality/Quantity, *F*(2,28) = 19.92, *p* < .001, η^2^_p_ = .585, with an increase from week 1 to week 2, *t*(15) = 2.28, *p* = .038, *d* = .57, and a further increase from week 2 to 3, *t*(15) = 4.20, *p* = .001, *d* = 1.05; Looking at Infant, *F*(2,28) = 19.76, *p* < .001, η^2^_p_ = .59, with greater looking in week 3 compared to either weeks 1 or 2, *t*(15) > 4.55, *p*s ≤ .001, *d*s > 1.14, but no difference between weeks 1 and 2, *p* = .169; Mutual Gaze, *F*(2, 28) = 7.72, *p* = .002, η^2^_p_ = .36, with an increase from week 1 to 2, *t*(15) = 3.87, *p* = .002, *d* = .97, but no difference between weeks 2 and 3, *p* = .091; Assess Hunger/Feed, *F*(2,28) = 273.84, *p* < .001, η^2^_p_ = .951, with a decrease from week 1 to 2, *t*(15) = 2.95, *p* = .011, *d* = .74, and a further decrease from week 2 to 3, *t*(15) = 20.86, *p* < .001, *d* = 5.22; Play, *F*(2,28) = 5.48, *p* = .010, η^2^_p_ = .281, with an increase from week 1 to 2, *t*(15) = 3.33, *p* = .007, *d* = .83, but no change between weeks 2 and 3, *p* = .122; Stroke/Pet, *F*(2,28) = 126.51, *p* < .001, η^2^_p_ = .900, with the most stroking in week 3, *t*(15) > 10.99, *p*s < .001, *d*s > 2.75, and a decrease in stroking from week 1 to week 2, *t*(15) = 3.66, *p* = .003, *d* = .92; Stimulate Vestibular, *F*(2,28) = 16.93, *p* < .001, η^2^_p_ = .547, with more stimulation in week 3 than in weeks 1 and 2, *t*(15) > 4.18, *p*s = .001, *d*s = 1.05, and no difference between weeks 1 and 2, *p* = .215; Sooth Infant, *F*(2,28) = 6.02, *p* = .007, η^2^_p_ = .301, with an increase in soothing from week 2 to 3, *t*(15) = 2.83, *p* = .013, *d* = .71, but no difference between the first two weeks, *p* = .067; Hold Infant, *F*(2,28) = 177.21, *p* < .001, η^2^_p_ = .927, with more holding in week 3 compared to weeks 1 and 2, *t*(15) > 13.32, *p*s < .001, *d*s > 3.33, and no difference between the first two weeks, *p* = .282. There were no other effects, *p*s > .05. Together, these results suggest that caretaking behaviors change as infants grow more independent with age, but do so in the same ways for female and male infants. In conclusion, while we cannot generalize these findings to other contexts, we think these observations suggest that the specific training protocols at this facility were effective in preventing caregiver bias.

References

1. Thomsen, C. E. Eye contact by non-human primates toward a human observer. *Anim. Behav.* **22**, 144-149. (1974).
2. Schino, G., Troisi, A., Perretta, G. & Monaco, V. Measuring anxiety in nonhuman primates: effect of lorazepam on macaque scratching. *Pharmacol. Biochem. Behav.* **38**, 889-891. (1991).

Supplementary Tables

*Table S1*. Human interaction task composite means (*M*) and standard deviations (*SD*) and interobserver reliability, assessed with correlations and paired-sample *t* tests (two-tailed).

*Table S2*. Human interaction task descriptive statistics for individual behaviors: means (*M*), standard deviations (*SD*), and paired-sample *t* tests (two-tailed) comparing males and females.

*Table S3.* Quality Assessment data of caregiver-infant observation means (*M*) and standard deviations (*SD*) for female and male infants across the first three weeks of life.

Supplementary Videos

*Video S1*. Video stimulus of animated adult monkey (head and shoulders) making eye-contact and producing a lipsmack (LPS) expression, a positive, affiliative gesture. An example of an infant’s fixations are shown in red (21-day-old female). This video stimulus was created by the third author, Melissa Shetler.

*Video S2*. Video stimulus of animated adult monkey (head and shoulders) making eye-contact and producing a fear grimace expression. An example of an infant’s fixations are shown in red (24-day-old male). This video stimulus was created by the third author, Melissa Shetler.

*Video S3*. Video stimulus of animated adult monkey (head and shoulders) making eye-contact and producing a threat expression. An example of an infant’s fixations are shown in red (11-day-old female). This video stimulus was created by the third author, Melissa Shetler.
